# Correlation of Proton Pump Inhibitors with Pulmonary Tuberculosis: A Case-Control Study in Taiwan

**DOI:** 10.3389/fphar.2017.00481

**Published:** 2017-07-19

**Authors:** Kao-Chi Cheng, Kuan-Fu Liao, Cheng-Li Lin, Shih-Wei Lai

**Affiliations:** ^1^College of Medicine, China Medical University Taichung, Taiwan; ^2^Department of Family Medicine, China Medical University Hospital Taichung, Taiwan; ^3^Department of Internal Medicine, Taichung Tzu Chi General Hospital Taichung, Taiwan; ^4^College of Medicine, Tzu Chi University Hualien, Taiwan; ^5^Graduate Institute of Integrated Medicine, China Medical University Taichung, Taiwan; ^6^Management Office for Health Data, China Medical University Hospital Taichung, Taiwan

**Keywords:** pulmonary tuberculosis, proton pump inhibitors, Taiwan National Health Insurance Program

## Abstract

**Background and Objectives:** Although the relationship between the use of proton pump inhibitors (PPIs) and pulmonary tuberculosis (TB) in Taiwan published in 2014. Due to just only one article and not enough comprehensively, we explore this issue.

**Methods:** We conducted a population-based case-control study to identify 9,422 subjects aged 20 years or older with newly diagnosed pulmonary TB in 2000–2013 as test cases. We then randomly selected 9,422 subjects aged 20 years or older without pulmonary TB as controls. Both cases and controls were matched in terms of sex, age, and comorbidities. Use of PPIs were defined as subjects who had had at least one prescription for these medications before the index date. No use was defined as subjects who had never had a prescription for PPIs before the index date. The odds ratio (OR) and 95% confidence interval (CI) for pulmonary TB associated with PPI use was estimated using the logistic regression model.

**Results:** The OR of pulmonary TB was 1.31 for subjects who had used PPIs (95% CI 1.22, 1.41) compared with those with no use of the medications. Sub-analysis revealed the OR of pulmonary TB in subjects using PPI per increasing microgram was 1.25 (95% CI 1.19, 1.30).

**Conclusions:** PPI use is associated with a 1.3-fold increase in odds of developing pulmonary TB in Taiwan. There is a dose-related response between PPI use and pulmonary TB.

## Introduction

Gastric acid plays a major role in decontaminating the upper gastrointestinal tract. A review article published about 30 years ago sought to determine the relationship between gastric secretion and ingested organisms resulting in possible infection (Howden and Hunt, 1987). Due to progress and recent advances in clinical pharmacology, acid-suppressive agents, including proton pump inhibitors (PPIs), have seen widespread use for treating peptic ulcers and acid reflux esophageal disease (Noguerado et al., 2002; Strid et al., 2003). Over the last 2–3 decades, PPIs were common used for acid suppressive agents in primary and specialty care with excellent safety (Sheen and Triadafilopoulos, 2011). A previous article revealed that PPIs appeared to be more effective than histamine 2 receptor antagonists (H2RAs) in preventing clinically important and overt upper gastrointestinal bleeding (Alhazzani et al., 2013).

Previous studies have described the relationship between PPI use and several enteric infections, such as *Salmonella enteritis* and *Clostridium difficile* colitis (Dial et al., 2005; Rodríguez et al., 2007), including spontaneous bacterial peritonitis in severe cirrhotic patients (Bajaj et al., 2009). Several research works have indicated that, besides the gastrointestinal system, PPIs are positively associated with infections of the respiratory system, such as community- or hospital-acquired pneumonia (Gulmez et al., 2007; Sarkar et al., 2008; Jager et al., 2012). However, few studies have indicated whether this association was related to low-dose or short-term PPI use (Giuliano et al., 2012; Filion et al., 2013). In addition to hospital- or community-acquired pneumonia, *Mycobacterium tuberculosis* (TB)-associated infection exerts significant burdens on the health-care systems of developing countries, including Taiwan (Hsueh et al., 2006).

Previous articles discussing the association between pulmonary TB and any degree of gastrectomy are scarce, and most of them do not include up-to-date technologies and true mechanism (Boman, 1956; Thorn et al., 1956). To date, the real role of gastric acid in pulmonary TB patients remains unknown. Although the relationship between the use of PPIs and pulmonary tuberculosis (TB) in Taiwan, similar to our study, published in 2014 (Hsu et al., 2014). Due to just only one article and not enough comprehensively (just focused on prescription period of PPIs only), we utilized the Taiwan National Health Insurance Program database to plan and conduct this study for exploring the associations completely and definitely.

## Methods

### Data source

Taiwan is an independent country with a population of over 23 million (Chao et al., 2015; Chen et al., 2015; Ho and Chang, 2015; Hsiao et al., 2015; Hung and Ku, 2015; Lin and Lin, 2016; Lin et al., 2016a; Maa and Leu, 2016; Ooi, 2016; Yu et al., 2016). We conducted a population-based case-control study using data from the Taiwan National Health Insurance Program. This insurance program was established in March 1995 and covers 99% of Taiwan's population (National Health Insurance Research Database, 2017). Details of this program can be found in previous studies (Lai et al., 2010, 2012; Hung et al., 2011; Cheng et al., 2012; Tsai et al., 2016). The present study was approved by the Research Ethics Committee of China Medical University (CMUH-104-REC2-115).

### Participants

We identified subjects aged 20 years or older with newly diagnosed pulmonary TB (International Classification of Diseases, Ninth Revision, Clinical Modification, ICD-9 codes 010, 011, 012, and 018) from 2000 to 2013 as test cases. The date of pulmonary TB diagnosis was defined as the index date. Subjects who were not diagnosed with pulmonary TB were randomly selected from the same database as controls. Both cases and controls were matched in terms of sex, age (5-year intervals), and comorbidities.

### Comorbidities potentially related to pulmonary PT

Comorbidities that could potentially be related to pulmonary TB, including alcohol-related diseases, asbestosis, chronic kidney disease, chronic obstructive pulmonary disease, diabetes mellitus, human immunodeficiency virus infection, gastrectomy, pneumoconiosis, splenectomy, and chronic liver diseases, such as cirrhosis, hepatitis B infection, hepatitis C infection, and other forms of chronic hepatitis, were assessed. All comorbidities were diagnosed with ICD-9 codes. The accuracy of these codes has been examined in previous studies (Lai et al., 2013a,b, 2014a,b, 2017; Hung et al., 2016; Lai, 2016; Lin et al., 2016a, 2016b; Shen et al., 2016; Hsu et al., 2017; Liao et al., 2017a,b).

### Measurements of PPI and H2RA use

The PPIs available in Taiwan between 2000 and 2013 and considered in this study included esomeprazole, lansoprazole, omeprazole, pantoprazole, and rabeprazole. Patients' prescription histories of PPIs and H2RAs were included in this study. Use of medications was defined as prescription any of the medications studied in this work before the index date. No use of medication was defined as no history of prescription of any of the medications studied in this work before the index date.

### Statistical analysis

We compared the distributions of demographic status, PPI use, H2RA use, and comorbidities between cases and controls using the chi-squared test for categorized variables. The *t*-test was used to test differences in mean age and mean duration of exposure to PPIs between cases and controls. The univariable unconditional logistic regression model was used to measure odds ratios (ORs) and 95% confidence intervals (CIs) and determine the association between pulmonary TB and PPI use. We also analyzed dose-related responses to PPI use. All analyzes were performed using SAS software (version 9.2; SAS Institute, Inc., Cary, NC, USA), and results were considered statistically significant when two-tailed *P*-values were < 0.05.

## Results

### Characteristics of the study population

As shown in Table 1, we identified 9,422 cases with newly diagnosed pulmonary TB between 2000 and 2013 and 9,422 controls without the disease. Both cases and controls showed similar distributions of sex and age. The mean ages (standard deviation) of patients with and without primary TB were 59.6 (17.1) years and 59.5 (17.1) years, respectively, and these values did not show statistical significance (*t*-test, *P* = 0.8). The mean durations of PPI use (standard deviation) of cases and controls were 3.19 (5.96) and 3.49 (6.06) months, respectively; these values did not show statistical significance (*t*-test, *P* = 0.13). While cases with pulmonary TB were more likely to report PPI use than controls (22.3 vs. 18%, chi-square test, *P* < 0.001), no significant difference in H2RA use or other comorbidities was observed between cases and controls (Chi-square test, *P* > 0.05).

**Table 1:** Information and comorbidities between pulmonary tuberculosis cases and controls.

**Variable**	**Non-tuberculosis** ***N*** = **9422**		**Tuberculosis** ***N*** = **9422**		*P*-value^*^
	***n***	**%**	***n***	**%**	
Sex					0.88
Female	3,000	31.8	3,010	31.9	
Male	6,422	68.2	6,412	68.1	
Age group (years)					0.99
20–39	1,551	16.4	1,549	16.4	
40–64	3,474	36.9	3,472	36.9	
65–84	4,397	46.7	4,401	46.7	
Age (years), mean (standard deviation)^†^	59.5	(17.1)	59.6	(17.1)	0.8
Duration of exposure to proton pump inhibitors (months), mean (standard deviation)^†^	3.49	(6.06)	3.19	(5.96)	0.13
Ever use of proton pump inhibitors	1,694	18	2,102	22.3	<0.001
Ever use of histamine-2 receptor antagonists	371	3.94	346	3.67	0.34
**COMORBIDITIES**					
Alcohol-related disease	717	7.61	745	7.91	0.45
Asbestosis	1	0.01	1	0.01	0.99
Chronic liver disease	1,637	17.4	1,674	17.8	0.48
Chronic obstructive pulmonary disease	4,111	43.6	4,136	43.9	0.71
Chronic kidney disease	459	4.87	468	4.97	0.76
Diabetes mellitus	1,433	15.2	1,477	15.7	0.38
Human immunodeficiency virus infection	21	0.22	21	0.22	0.99
Gastrectomy	9	0.1	9	0.1	0.99
Pneumoconiosis	87	0.92	75	0.80	0.34
Splenectomy	2	0.02	2	0.02	0.99

*Data are presented as the number of subjects in each group with percentages given in parentheses, or mean with standard deviation given in parentheses*.

* Chi-square test, and

† *t-test comparing subjects with and without pulmonary tuberculosis*.

### Pulmonary tuberculosis associated with proton pump inhibitor use

In Table 2, analyzes using the univariable unconditional logistic regression model showed that that the OR of pulmonary TB was 1.31 for subjects who had used PPIs (95% CI 1.22, 1.41) compared with subjects who had never used PPIs. Because no other variable was significantly related to pulmonary TB during univariable analysis, we did not perform multivariable unconditional logistic regression analysis.

**Table 2:** Odds ratio and 95% confidence interval of pulmonary tuberculosis associated with proton pump inhibitors use and comorbidities.

**Variable**	**Crude**	
	**OR**	**(95%CI)**
Sex (male vs. female)	1.00	(0.94, 1.06)
Age (per 1 year)	1.00	(0.99, 1.00)
**PROTON PUMP INHIBITORS (NEVER USE AS A REFERENCE)**		
Ever use	1.31	(1.22, 1.41)
**HISTAMINE-2 RECEPTOR ANTAGONISTS (NEVER USE AS A REFERENCE)**		
Ever use	0.93	(0.80, 1.08)
**COMORBIDITIES (YES VS. NO)**		
Alcohol-related disease	1.04	(0.94, 1.16)
Asbestosis	1.00	(0.06, 16.0)
Chronic liver disease	1.03	(0.95, 1.11)
Chronic obstructive pulmonary disease	1.01	(0.95, 1.07)
Chronic kidney disease	1.02	(0.89, 1.17)
Diabetes mellitus	1.04	(0.96, 1.12)
Human immunodeficiency virus infection	1.00	(0.55, 1.83)
Gastrectomy	1.00	(0.40, 2.52)
Pneumoconiosis	0.86	(0.63, 1.17)
Splenectomy	1.00	(0.14, 7.10)

*CI: confidence interval*.

### Sub-analysis of the association between pulmonary tuberculosis and five proton pump inhibitors

Table 3 shows an analysis of the dose-related responses to PPI use; here, the group with no PPI use was considered the reference group. The OR of pulmonary TB in subjects using PPI per increasing microgram was 1.25 (95% CI 1.19, 1.30). These results indicate a dose-related response between PPI use and pulmonary TB.

**Table 3:** Odds ratio and 95% confidence interval of pulmonary tuberculosis in relation to cumulative dosage of proton pump inhibitors use by logistical regression model.

**Variable**	**Case number/control number**	**Crude OR**	**(95% CI)**
Never use of proton pump inhibitors as a reference	7320/7728	1.00	(reference)
Proton pump inhibitors use (increase in dosage per mg)	2102/1694	1.25	(1.19, 1.30)

*CI: confidence interval*.

## Discussion

PPIs have been commonly prescribed to patients with upper gastrointestinal tract bleeding or acid reflux-associated diseases in the last two decades (Hsu et al., 2014). These medications feature an excellent safety profile, and their benefits outweigh their risks in most patients worldwide (Sheen and Triadafilopoulos, 2011). Patients began using PPIs for extended periods of time without considering appropriate indications for primary and specialty care. This problem has intensified in Taiwan, especially in the last two decades (Chen et al., 2003). Achlorhydric stomach is usually accompanied by a weak protective mechanism for ingested organisms, which could bring about gastrointestinal (Howden and Hunt, 1987; Williams and McColl, 2006) and respiratory infections, such as hospital- or community-acquired pneumonia.

Although previous published article in 2014 associated with Taiwan general PPIs user populations and pulmonary TB patients had similar study design and article structure compared with ours (Hsu et al., 2014). Their result revealed that the relationship between PPIs and pulmonary TB patients became gradually faded when the drug prescription period extended. But in our study, we found that cases with pulmonary TB were more likely to have used PPIs only than controls without the disease. General speaking, the relationship between PPIs (accumulative dose) and pulmonary TB is positive in our study, but negative in theirs (accumulative time). Because of the different results in prescription period or accumulative dosage between both of them, we planned and conducted our study for further investigation. This finding in our study may be explained by several hypotheses as follows.

First, long-term PPI use may weaken the protective mechanism of the stomach because PPIs inhibit gastric secretion. While the acidic environment of the stomach is generally free from bacteria (Vakil, 2009), bacterial colonies gradually develop and increase in achlorhydric or hypochlorhydric stomachs. Frequent and long-term use of PPIs is believed to promote infections in patients prescribed these medications.

Second, the possible explanation might be associated with lower immune status. *M. tuberculosis* shares risk factors similar to those of community-acquired pneumonia, such as diabetes mellitus, alcohol drinking, aging, and HIV infection (Hsu et al., 2014), although our study showed no significant difference in these comorbidities between cases and controls. A recent large-scale study in Japan and Taiwan revealed that gastrectomy could be associated with increased risk of pulmonary TB (Yokoyama et al., 2004; Huang et al., 2011), leading to poor immunity and nutritional states. We can confirm that the mechanism of gastrectomy is similar to that of PPI use and results in an achlorhydric or hypochlorhydric stomach. Therefore, the risk of developing pulmonary TB may increase because of limited stomach acid secretion and the poor immune status of a patient.

Finally, physicians in hospitals or local clinics may have difficulty in differentiating the symptoms of the initial stages of pulmonary TB and those of acid reflux-associated diseases, such as dry or chronic cough. The patients with chronic cough, disregarding whether pulmonary TB infections or not might be misdiagnosed or ignored, then PPI medication prescribed for treating acid reflux-associated chronic cough by physicians belong reasonable inference. Thus, some patients may still be infected with pulmonary TB even after PPI therapy. On the other words, PPI therapy should be withheld from patients with highly suspect pulmonary TB, even not prescribed PPI before confirm diagnosis of pulmonary TB.

In this study, we analyzed dose-related responses to PPI use and considered controls with no PPI use as the reference group. The dose-related response is understandable (Chou and Talalay, 1984). Patients prescribed high doses of the medications showed more extensive adverse effects than those prescribed relatively lower doses (Sheen and Triadafilopoulos, 2011). As PPI use is gradually increasing in Taiwan (Chen et al., 2003), physicians and specialists should pay more attention to the dose-related responses to and adverse effects of PPIs.

## Limitation

One of the limitations of this work is underestimation of the numbers of patients with PPI use. Herein, we focused only on Taiwan's National Health Insurance Program database and ignored PPI or H2RA medications prescribed over the counter. Another limitation is diagnosis of pulmonary TB using ICD-9 codes. Miscoding by physicians, an ambiguous definition of pulmonary TB, and lack of sputum culture or chest X-ray could contribute to misdiagnosis of the disease. Finally, history, period, and dosage of PPI therapy were defined arbitrarily and without following any standard guideline. Rigorous evaluation of the definitions of pulmonary TB and manner of PPI use is recommended for future investigations.

## Strength

One of the strengths of the present study is that the set of ICD-9 codes used has been validated in previous published studies (Lai et al., 2013a,b, 2014a,b, 2017; Hung et al., 2016; Lai, 2016; Lin et al., 2016a, 2016b; Shen et al., 2016; Hsu et al., 2017; Liao et al., 2017a,b). The long observation period employed in our study (i.e., from 2000 to 2013) also endows our study with more credibility compared with other similar studies.

## Conclusion

We conclude that PPI use is associated with a 1.3-fold increase in odds of developing pulmonary TB in Taiwan. There is a dose-related response between PPI use and pulmonary TB. Considering our results, evaluation of the risk of developing pulmonary TB may be necessary prior to prescribing the use of anti-suppressive agents.

## Author contributions

KC and KL planned and conducted this study. They participated in the data interpretation, and also critically revised the article. CL conducted the data analysis and critically revised the article. SL planned and conducted this study. He contributed to the conception of the article, initiated the draft of the article, and critically revised the article.

### Conflict of interest statement

The authors declare that the research was conducted in the absence of any commercial or financial relationships that could be construed as a potential conflict of interest.
